# Societal perspectives on risk awareness and risk competence

**DOI:** 10.3205/000212

**Published:** 2015-07-09

**Authors:** Michael Koller, Ulrich Hoffrage

**Affiliations:** 1 Centre for Clinical Studies, University Hospital Regensburg, Germany; 2 Faculty of Business and Economics, University of Lausanne, Switzerland

**Keywords:** freedom of choice, safety, risk society, decision-making processes, risk communication, risk minimisation, information culture

## Abstract

Medical risks can be assessed by objectifiable therapeutic features; however, these risks are also characterised to a considerable degree by individual and social values. People tend to strive towards both freedom as well as safety; in a medical context, these two aims are taken into account by shared decision-making models and by stricter regulations in the pharmaceutical sector. Media reports on medical risks are caught between providing information and economic interests, and this conflict particularly complicates rational discussions about unexpected risks (for instance, in the field of natural medicine). Thus, it is necessary to create the type of information culture which allows differentiating between real and less pronounced risks.

## Zusammenfassung

Risiken innerhalb der Medizin sind nicht bloß durch objektivierbare Eigenschaften von Therapien fassbar, sondern in erheblichem Maße durch individuelle und gesellschaftliche Werte geprägt. Individuen streben gleichermaßen nach Freiheit und Sicherheit – im medizinischen Kontext wird dem durch partizipative Entscheidungsmodelle einerseits und Verschärfungen der Regularien im Arzneimittelsektor andererseits Rechnung getragen. Die mediale Berichterstattung über Risiken im Gesundheitsbereich findet im Spannungsfeld zwischen Aufklärung und wirtschaftlichen Interessen statt, was insbesondere eine rationale Diskussion über unerwartete Risiken (z.B. in der Naturheilkunde) erschwert. Es gilt daher eine Informationskultur zu schaffen, die es erlaubt, echte von weniger ausgeprägten Risiken zu unterscheiden.

## Individuals and society in the conflict between freedom and security

The freedom of choice is a fundamental motivational aim of people [1]. Thus, patronizing behaviour by society or specific policy makers is poorly accepted. 

In medicine, such freedom consists of the patients’ desire for self-determination. In the classic paternalistic decision model, physicians are viewed as omniscient, sometimes even as omnipotent agents (demi-gods in white), who determine the treatment and therapy of patients as experts [2]. In our modern information society marked by the permanent availability of medical knowledge, this paternalistic model is no longer up-to-date and thus becoming increasingly replaced by collaborative decision-making processes. Shared decision-making is characterised by the exchange of information between patients and physicians and results in decisions supported by both parties [2]. 

Evidence-based medicine plays its part in challenging the authority of physicians [3]. Paternalistic societies viewed the expert opinion of physicians as binding medical truth and as a guiding principle of medical practice. Modern societies, however, demand scientific evidence proven in clinical studies. According to the classification developed by the Oxford Centre for Evidence-based Medicine (OCEM; Table 1 (Tab. 1)), the highest degree of evidence is provided by randomised controlled clinical studies or, even better, by meta-analyses of several high-quality randomised trials [3]. The lowest degree of evidence is attributed to expert knowledge, ranging even below observational studies and case series. The excessively used term ‘paradigm shift’ is fully justified in this case [4]. 

A special, sometimes even dangerous form of searching for freedom can be found in the leisure sector. Recreational activities are often based on fun and adventure, which are increased by mastering a potentially dangerous situation. True to the motto: No risk, no fun. In psychology, such practices are known as sensation seeking behaviour [5]. Nevertheless, – and here it starts getting complicated –, individuals expect solidarity from other people. The strive for freedom and the finely metered search of danger is viewed and defended as a private good – but in case of an accident or an emergency, the help by others is demanded as a matter of course. (This behaviour can also be observed in other areas: Banks are allowed to speculate and make huge profits. If things go wrong, the tax payers have to bail them out.) Society feels responsible for ensuring the safety of its members but also for controlling the behaviour of its members and to minimise their risks. Social institutions and instruments contribute to the increase in safety: Emergency services, hospitals, police and many more. Such services are funded by health insurance contributions and taxes. People who pay contributions feel they have the right to benefit from such social instruments.

In order to prevent damages occurring in the first place, society developed instruments for behaviour control, such as laws, prohibitions and social norms. Wiedemann [6] analysed the development of these instruments from a historical perspective. According to his analysis, the concept of risk appeared relatively late in the history of civilisation – its predecessor was the concept of danger. ‘Dangers are imposed from without, while risks are incurred‘ ([6], p. 43). In ancient times, reasoning and visualising included rather vivid pictures, and nature was seen as animated. In order to protect people from specific external dangers, certain forms of behaviour became taboo, and such taboos were followed without questioning. Wiedemann views the development of religions and their assumedly divine regulations as the next step. Revolt against these systems is possible but seen as sin. Thus, the concept of sin is an inevitable part of the freedom to participate in decision-making processes. Sin meant isolation and disentanglement from God’s plan and order, in turn leading to diverse punishments, both in this world and in the hereafter. After the decreasing influence of religious conceptions and the increasing importance of natural sciences and technology, the aim of striving for a life pleasing unto God is replaced by maximising benefits in this world. According to Wiedmann, behaviour is not determined by God's commandments and prohibitions anymore but a calculated consequence that may result in taking a certain risk – simply because the expected benefit is higher than the anticipated damage. 

In the course of human development, our lives have become more secure, which can be easily seen in the increase in life expectancy. Centuries ago, sudden infant death was accepted as God’s will and as being tested or punished. Medical improvements and the discovery of causal chains changed the conception of this cause of death from being God’s will to a medical error made by physicians or midwifes. Steps needed to be taken in order to ensure that such deaths would not occur again. The more relevant factors could be controlled, the higher became the demand for further increasing safety. This way, the topic risk and safety became omni-present [7]. 

## Risk society

The construct of risk is a downright characteristic of modern society and has considerable implications regarding behaviour and economics. The risk analysis by Beck is widely known in this context [8]. Beck understands the term ‘risk’ as ‘scientific distribution of pollutants’ as well as a ‘social risk situation’, for instance, unemployment ([8], p. 31). A characteristic feature of this concept is that respective risks are not distributed according to social class anymore. On the contrary, risks can potentially affect anybody, for instance, radioactive radiation would not distinguish between rich and poor: ‘Poverty is hierarchic, smog is democratic’ ([8], p. 48). Beck states that risks are also always the result of social construction processes. It is not the abstract risk itself that is perceived as a danger but its being addressed by the mass media, which results in ‘reality […] being cognitively structured and perceived according to a schema of safety and danger’ ([8], p. 48).

Therefore, media information on risks significantly contribute to this process. The more abstract the communicated danger, the higher is the perception of the loss of control. As a defence strategy, risks become inflated. Because of their different levels of knowledge, the risk assessment of experts and laypeople often differs [9], which results in different levels of risk inflation.

## Risk in political decision-making processes

In 1988, Freudenburg investigated the extent to which laypeople may rely on experts [10]. Laypeople tend to have little experience with regard to abstract risks or new technologies (for instance, genetically modified foods, nanotechnology or fracking) and have very limited access to information necessary for risk assessment. This situation is aggravated by the fact that no long-term studies are (and cannot be) available, particularly on new technologies. Experts often know more about the topic but have a reputation of being influenced by the commercial interests of the industry. The people’s need for security seems to be of minor importance. Who is going to decide now? The politicians? Are they able to arbitrate between experts and laypeople?

The precautionary principle states that new technologies should not be introduced in the case of any doubts [11]. The application of this principle in political decisions reverses the burden of proof: Not the opponents have to prove that a new technology is harmless but the supporters have to provide evidence that the technology does not bear any risk, ideally under the eyes of an aware and critical public.

Technological progress can only be achieved by entering new territory, which will always involve uncertainties and risks. Benefits and risks can hardly be determined without any sound knowledge or empirical values. Today, people use fast trains all the time and laugh about the sceptics who warned about health risks due to the ‘high speed’ of 28 km/h of the first train running from Nuremberg to Fuerth in 1835. On the other hand, – after Chernobyl and Fukushima – nobody laughs about people warning about the dangers of the civilian use of nuclear energy. The problem is that we will only know in hindsight if a new technology will bring progress or further evil creeping out of Pandora’s box.

## Balanced risk

Improving products with regard to design, material properties and safety usually leads to the preference of new products over their predecessors. This simple observation fully complies with Lübbe’s analysis. But what do people do with this newly gained safety? Such safety is often ruined again by people’s behaviour. This is the main message of the risk homeostasis theory: People accept a certain degree of risk [12]. If risk is reduced by certain measures, for instance, by better brakes in cars or by wearing a safety helmet on bicycles, people do not enjoy the achieved increase in safety but compensate such measures by driving even faster. This theory is in line with the above-mentioned concept of benefits calculation. The benefit of increased safety is converted into another benefit – here arriving earlier at the destination. The aim of engineers of making products safer is not counteracted if additional safety features are not observable or perceivable. Better brakes ‘invite’ people to drive faster, shatterproof windscreen do not [13].

Even if the term ‘risk’ has a rather negative connotation in German and is often equated with danger in everyday life, risk taking can indeed be rather beneficial. The benefit may be in areas which are rather disregarded in the first place: Risky decisions may have a positive influence on social communities and contribute to the building of trust. Examples can be found in studies from the field of experimental social psychology [14]. In an experiment, study subjects were required to lend a book to another person. One experimental condition involved a rather inexpensive book (low damage and thus a small risk in the case of loss) and another condition an expensive book (high risk). After having made a decision, the study subjects had to state whether they would also trust their interaction partners in other situations. The experiment showed that trust was more pronounced in the case of high risk than in the case of low risk.

Viewed in this light, risk is an important social bond which strengthens cohesiveness in a group. This is the only explanation for being able to establish trust with physicians even in difficult and stressful situations and for ensuring the flow of social interaction. However, there are limitations. Risks are accepted and make a positive social contribution as long as they appear controllable or as long as safety features are available [15].

## Risk communication in the media using the example of natural medicines

In general, medicine and health care systems are among the most popular media topics. The high importance and the increasing sensibility for safety as a commodity generates good business for media reports on risks: ‘(Only) bad news is good news’. The combination of medicine and risk is especially volatile. Negative media reports receive a particularly high level of attention if they concern drugs that are originally considered safe. Thus, media reports on natural medicines are of particular importance. Natural medicines have a positive image and are the epitome of ‘gentle’ medicine. Reports on the danger of such medicines considered safe and natural receive a high level of attention. On a list compiled by the Hessian Broadcasting Corporation in July 2013, natural medicines referred to as ‘Valerian, St. John’s wort and others’ can be found on position 6 of the most dangerous toxins in households [16] (Table 2 (Tab. 2)). 

The ranking order within this list was probably significantly influenced by the varying media reports on the health risks of natural medicines. St. John’s wort, for instance, which is proven to relieve mild to moderate depression [17], is associated with impeding the effectiveness of contraceptives [18]. Herbal supplements containing the kava kava root have proven their effectiveness as an anxiety-reducing drug, both in clinical studies and in daily practice [19], [20]. In 2002, however, the German Federal Ministry for Pharmaceuticals and Medical Products withdrew the approval for herbal supplements containing kava kava because of some cases of liver failure assumed to have been associated with the intake of these supplements. Subsequent studies and analyses called this association into question, but the ban was not revoked [21]. At present, it is vitamin products which are under attack [22]. Not only is the effectiveness of vitamins being questioned, but the intake of highly dosed multivitamin preparations is suspected to have adverse health effects and may even cause cancer [23].

Although the role of the media is of central importance for an informed society, the tendency of generalising judgements becomes particularly clear in this connection. Such generalisations are marked by the following patterns:

- It is not reported that data are uncertain.
- It is not shown that different preparations have different effects: Discount tea preparations cannot be compared with special extracts produced by the pharmaceutical industry, even if the raw material is the same.
- It is not shown how the risk profile of natural medicines compares with that of conventional medications.
- The problem of overdosing and uncontrolled intake over a long period of time is not discussed.
- Influences of the underlying condition of the patient and the interaction with the intake of other medications (polymedication) are disregarded.

Without any doubt, such generalising media reports do not help to rationally assess the possible risk of natural medicines. 

The two-fold nature of the topic risk and safety and the associated public representation of natural medicines become particularly apparent in the context of homeopathy. Opponents of homeopathy regard such medicines as ineffective because they do *not* involve any risks. In Britain, a movement has developed who organise awareness-raising activities under the heading *‘There’s nothing in it’* [24]. In the best of British traditions – please note the parallel to the Atheist Bus Campaign *‘There probably is no God. Now stop worrying and enjoy your life’* [25], [26] –, activists publicly swallow lots of homeopathic medicines, preferentially in front of pharmacies, without dropping dead. The non-occurrence of adverse effects is seen as a proof for the ineffectiveness of homeopathic medicines.

Advocates of homeopathy may be accused of not providing sufficient evidence for the efficacy of homeopathic medicines. Nevertheless, the above-mentioned activists make a mockery of a basic rule of evidence-based medicine: *‘Absence of evidence is not evidence of absence’* [3]. In other words, the absence of serious adverse effects or death does neither prove the general ineffectiveness of a particular group of medicines nor the incorrectness of a school of thought. Would the same activists also assemble in front of a chemist shop, wash their hair for hours and – after the non-occurrence of serious adverse effects – believe that they have proven the ineffectiveness of shampoos? 

The controversy about homeopathy is a classic example of how the concept ‘risk’ arouses most people’s feelings while simultaneously invalidating some people’s logical thinking skills.

## Social mechanisms for minimising risk with regard to medicinal products

The regulations on medical research and clinical drug studies have been developed over many decades, from the foundation of the American Food and Drug Administration (FDA) in 1938 to the Declaration by the World Medical Association in 1964. The ICH-GCP (*International Conference on Harmonization – Good Clinical Practice*) guidelines were laid down as an internationally binding framework only in 1996 [27]. These regulations were established for two purposes: patient safety and data quality. Serious and tragic events in the context of developing and testing treatment measures (keyword ‘Contergan’) should be prevented this way.

In Germany, this conceptual stipulation was transposed into national law by the 12^th^ amendment of the Medicines Act including GCP regulations via the EU Directive 2001/20/EG in 2004. This law has made the application and realization of clinical trials substantially more stringent. By now, it is imperative that investigators and study personnel have to attend study-specific training programmes. Study centres are required to adhere to quality standards and obtain appropriate certifications. At the same time, the range of training programmes and services is steadily increasing. The spiral of supply and demand has developed its own dynamics and is fed by regular amendments of the regulations.

Interestingly, patient safety and data quality as the basic concepts of ICH-GCP have disappeared from view. Evidence has never been provided nor required that the continuous tightening of regulations increases safety. From the perspective of emotional psychology, an exactly opposite hypothesis can be established: Excessive regulating generates considerable threat potential, which leads to ‘tunnel vision’ [28], undermines the capability to separate important from unimportant issues and results in more mistakes. The chain of reasoning is coherent but needs to be rebutted the same way as the assumption that stricter regulations increase safety needs to be confirmed. However, the fact that the increase in regulations has inflated the costs of clinical studies is uncontested [29].

First corrections with regard to deescalating regulations can be observed. For the first time, empirical analyses within the ADAMON study shall show the importance of risk-adapted monitoring for the quality of clinical studies [30]. New EU directives, whose implementation is scheduled for 2016, aim at simplifying the approval of clinical studies at ethics committees and authorities, particularly with regard to multinational drug studies [31]. It remains to be seen whether these and other similar initiatives will truly result in sustainable improvements.

## Consequences and need for discussion

The keywords ‘safety and risk in health care’ relate to an extremely sensitive subject. This article has shown that the concepts of safety and risk are decisively shaped and conveyed by society and do not just exclusively characterize treatment properties. For a rational discussion, two statements are necessary:

1. Health care is marked by the interaction of both social and economic interests, which are often based on different objectives.
2. Similar tension is often found in the media sector: The socially so important task of informing the public is accompanied by economic interests served by flat, simplified and risk-enhancing media reports.

It is important to create an information culture which allows individual people to understand these different interests and to distinguish real risks from less pronounced risks. This topic is addressed by Hoffrage and Koller in a separate article [32] in this special issue.

## Notes

### Acknowledgement

The authors gratefully acknowledge the constructive comments by Kirsten Schaefer (psychologist).

### Competing interests

The authors declare that they have no competing interests.

## Figures and Tables

**Table 1:**
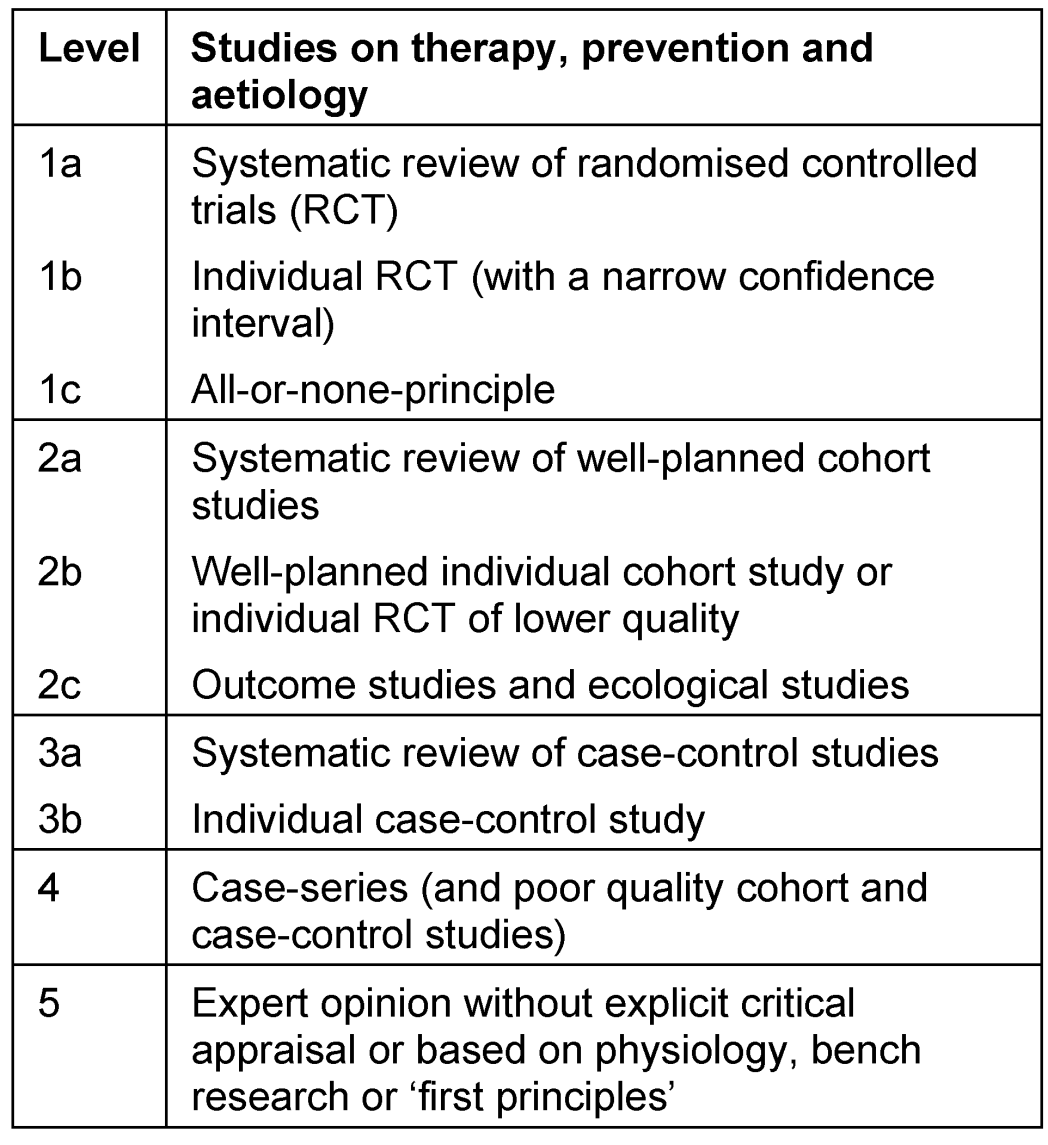
Classification of evidence levels (according to the Oxford Centre of Evidence-Based Medicine 2001)

**Table 2:**
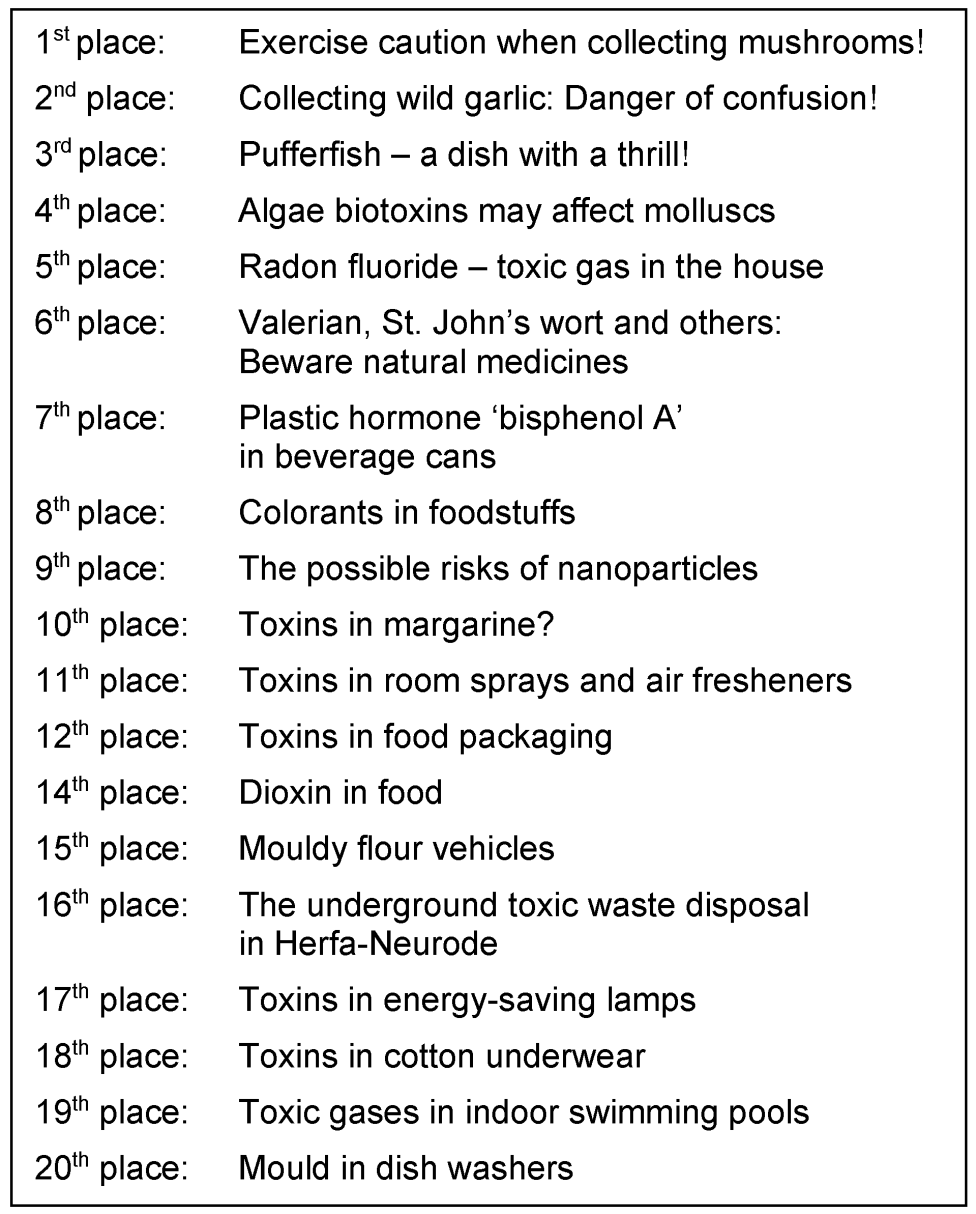
Everyday household dangers and risks (‘Knowing everything’, Hessian Broadcasting Corporation, July 2013)
